# Supplementary material for: Temperature explains broad patterns of Ross River virus transmission
Source: eLife. 2018 Aug 28;7:e37762. doi: 10.7554/eLife.37762 (PMC6112853; doi:10.7554/eLife.37762)
Supplement: Figure 1—source data 1. — Location and year of RRV disease outbreaks and mosquito species identified as likely vectors based on the collection of field specimens. Only the six most important mosquito species are included. Citation key: A: Biggs and Mottram, 2008; B: Brokenshire et al., 2000; C: Campbell et al., 1989; D: Dhileepan, 1996; E: Frances et al., 2004; F: Harley et al., 2000; G: Harley et al., 2001; H: Jacups et al., 2008b; I: Kelly-Hope et al., 2004b; J: Lau et al., 2017; K: Yang et al., 2009; L: Lindsay et al., 1993b; M: Lindsay et al., 1993a; N: Lindsay et al., 1996; O: Lindsay et al., 2007; P: McDonnell et al., 1994; Q: McManus et al., 1992; R: Merianos et al., 1992; S: Ritchie et al., 1997; T: Rosen et al., 1981; U: Russell et al., 1991; V: Russell, 1994; W: Russell, 2002; X: Ryan et al., 2000; Y: Schmaedick et al., 2008; Z: Whelan et al., 1992; AA: Whelan et al., 1995; AB: Whelan et al., 1997. [file elife-37762-fig1-data1.docx]

| **Location** | **Year(s)** | **Species** | **Citation(s)** |
| --- | --- | --- | --- |
| *Queensland* |  |  |  |
| Cairns | 1996-8 | *Ae. vigilax*  *Ae. notoscriptus*  *Cx. annulirostris* | F |
| Shoalwater Bay Training Area | 1997-8 | *Ae. vigilax*  *Cx. annulirostris* | I, E |
| Brisbane + surrounding area | 1994, 1996 | *Ae. vigilax*  *Ae. notoscriptus*  *Cx. annulirostris* | I, S, X |
| Warwick | 2004 | *Ae. vigilax* | A |
| *New South Wales* |  |  |  |
| Sydney suburbs | 1999 | *Ae. notoscriptus*  *Cx. annulirostris* | B |
| Bateman’s Bay | 1985-8 | *Ae. vigilax* | U |
| Southern inland areas | 1983-4, 1993 | *Cx. annulirostris* | G, I, P |
| *Victoria* |  |  |  |
| Gippsland region | 1988-9 | *Ae. camptorhynchus* | G, C, V |
| Murray River valley area | 1991-1995 | *Cx. annulirostris* | I, D |
| *South Australia* |  |  |  |
| Murray River | 1991-3 | *Cx. annulirostris* | I, V |
| *Western Australia* |  |  |  |
| Wheatbelt region | 1992 | *Ae. camptorhynchus* | O |
| Peel region + Bunbury | 1995-6 | *Ae. camptorhynchus*  *Ae. vigilax* | I, N |
| Perth | 1991-2 | *Ae. camptorhynchus* | G, K |
| Gascoyne region (coastal) | 1992 | *Ae. vigilax* | M |
| Pilbara region (coastal) | 1992 | *Ae. vigilax* | L |
| Kimberley region (inland) | 1982-6 | *Cx. annulirostris*  *Ae. normanensis* | M |
| *Northern Territory* |  |  |  |
| Darwin | 1991-2006 | *Ae. vigilax*  *Ae. notoscriptus*  *Cx. annulirostris* | H, Z, AB |
| Nhulunbuy | 1991-2 | *Ae. vigilax* | R, Z |
| Alice Springs + surrounding area | 1995 | *Cx. annulirostris*  *Ae. normanensis* | W, AA |
| *Tasmania* |  |  |  |
| Hobart + surrounding area | 1991 | *Ae. camptorhynchus* | G, Q |
| *Pacific Island nations* |  |  |  |
| American Samoa | NA | *Ae. polynesiensis* | J, Y |
| Cook Islands | 1981 | *Ae. polynesiensis* | T |

**Figure 1-source data 1**: **Vector species implicated in RRV disease outbreaks**. Location and year of RRV disease outbreaks and mosquito species identified as likely vectors based on the collection of field specimens. Only the six most important mosquito species are included.

Citation key: A: Biggs & Mottram 2008; B: Brokenshire et al. 2000; C: Campbell et al. 1989; D: Dhileepan 1996; E: Frances et al. 2004; F: Harley et al. 2000; G: Harley et al. 2001; H: Jacups et al. 2008; I: Kelly-Hope et al. 2004; J: Lau et al. 2017; K: Lindsay et al. 1992; L: Lindsay et al. 1993b; M: Lindsay et al. 1993a; N: Lindsay et al. 1996; O: Lindsay et al. 2007; P: McDonnell et al. 1994; Q: McManus et al. 1992; R: Merianos et al. 1992; S: Ritchie et al. 1997; T: Rosen et al. 1981; U: Russell et al. 1991; V: Russell 1994; W: Russell 2002; X: Ryan et al. 2000; Y: Schmaedick et al. 2008; Z: Whelan et al. 1992; AA: Whelan et al. 1995; AB: Whelan et al. 1997.
