# Supplementary material for: Temperature explains broad patterns of Ross River virus transmission
Source: eLife. 2018 Aug 28;7:e37762. doi: 10.7554/eLife.37762 (PMC6112853; doi:10.7554/eLife.37762)
Supplement: Figure 2—source data 1. — ‘Par.’=model parameter. Results are given for fits from data-informed priors. Asymmetrical responses fit with Brière function (B): B(T)= qT(T – Tmin)(Tmax – T)1/2; symmetrical responses fit with quadratic function (Q): Q(T) = -q(T – Tmin)(T – Tmax). Function coefficients (and 95% credible intervals) fit via Bayesian inference. [file elife-37762-fig2-data1.docx]

| **Par.** | **Definition** | **Species (Sources)** | **Fit** | **Function Coefficients (95% CIs) & Optimal Temperature** |
| --- | --- | --- | --- | --- |
| *a* | Biting rate = 1 / gonotrophic cycle duration (day)^-1^ | *Cx. annulirostris* (Russell 1986) | **B** | *T_min_* = 4.0 (0.7 – 8.5)  *T_max_* = 39.1 (37.9 – 40.0)  *q* = 1.26·10^-4^ (1.02 – 1.70·10^-4^)  optimum = 31.8°C |
| *bc* | Vector competence (transmission probability) | *Ae. vigilax* (Kay & Jennings 2002) | **Q** | *T_min_* = 6.0 (1.6 – 9.6)  *T_max_* = 42.7 (39.6 – 44.9)  *q* = 2.88·10^-3^ (2.02 – 4.27·10^-3^)  optimum = 24.4°C |
| *lf* = 1/*μ* | Adult lifespan (days) | *Cx. annulirostris* (McDonald *et al.* 1980) | **Q** | *T_min_* = 13.0 (10.6 – 14.6)  *T_max_* = 33.6 (33.0 – 34.5)  *q* = 0.241 (0.177 – 0.304)  optimum = 23.4°C |
| *PDR* | Parasite development rate (day)^-1^ | *Ae. vigilax* (Kay & Jennings 2002) | **B** | *T_min_* = 6.3 (1.3 – 13.0)  *T_max_* = 41.1 (36.1 – 45.0)  *q* = 1.38·10^-4^ (0.855 – 2.14·10^-4^)  optimum = 33.0°C |
| EFD | Fecundity (eggs per female per day) | *Cx. annulirostris* (McDonald *et al.* 1980) | **Q** | *T_min_* = 14.7 (11.9 – 17.2)  *T_max_* = 31.4 (30.3 – 32.9)  *q* = 5.84·10^-3^ (3.90 – 8.71·10^-3^)  optimum = 27.0°C |
| pRH | Raft viability (probability of raft hatching) | *Cx. annulirostris* (McDonald *et al.* 1980; Mottram *et al.* 1986) | **Q** | *T_min_* = 14.3 (10.6 – 17.2)  *T_max_* = 38.1 (33.7 – 42.0)  *q* = 6.78·10^-3^ (3.36 – 12.6·10^-3^)  optimum = 26.2°C |
| nLR | Within-raft egg survival (number of larvae per raft) | *Cx. annulirostris* (Mottram *et al.* 1986) | **Q** | *T_min_* = 17.0 (15.6 – 17.9)  *T_max_* = 37.2 (36.4 – 38.4)  *q* = 2.73·10^-3^ (1.99 – 3.29·10^-3^)  optimum = 27.0°C |
| *pLA* | Larval-to-adult survival (probability) | *Cx. annulirostris* (McDonald *et al.* 1980; Mottram *et al.* 1986; Rae 1990) | **Q** | *T_min_* = 14.9 (13.3 – 16.5)  *T_max_* = 39.1 (37.9 – 40.4)  *q* = 5.39·10^-3^ (4.31 – 6.65·10^-3^)  optimum = 27°C |
| *MDR* | Mosquito development rate (day)^-1^ | *Cx. annulirostris* (McDonald *et al.* 1980; Mottram *et al.* 1986; Rae 1990) | **B** | *T_min_* = 11.1 (7.6 – 14.4)  *T_max_* = 39.2 (37.9 – 40.1)  *q* = 6.86·10^-5^ (5.47 – 8.47·10^-5^)  optimum = 32.6°C |

**Figure 2-source data 1: Trait thermal response functions and data sources for Ross River virus *R_0_* models (eqs. 1 and 2).** ‘Par.’ = model parameter. Results are given for fits from data-informed priors. Asymmetrical responses fit with Brière function (**B**): B(*T*) = *qT*(*T* – *T_min_*)(*T_max_* – *T*)^1/2^; symmetrical responses fit with quadratic function (**Q**): Q(*T*) = -*q*(*T* – *T_min_*)(*T* – *T_max_*). Function coefficients (and 95% credible intervals) fit via Bayesian inference.
