# Supplementary material for: Temperature explains broad patterns of Ross River virus transmission
Source: eLife. 2018 Aug 28;7:e37762. doi: 10.7554/eLife.37762 (PMC6112853; doi:10.7554/eLife.37762)
Supplement: Figure 2—source data 2. — ‘Par.’=model parameter. Fits were made with uniform priors. Asymmetrical responses fit with Brière function (B): B(T)= qT(T – Tmin)(Tmax – T)1/2; symmetrical responses fit with quadratic function (Q): Q(T) = -q(T – Tmin)(T – Tmax). Function coefficients (and 95% credible intervals) fit via Bayesian inference. [file elife-37762-fig2-data2.docx]

| **Par.** | **Definition** | **Species (Sources)** | **Fit** | **Function Coefficients (95% CIs) & Optimal Temperature** |
| --- | --- | --- | --- | --- |
| *a* | Biting rate = 1 / gonotrophic cycle duration (day)^-1^ | *Anopheles pseudopunctipennis* (Lardeux *et al.* 2008); *Ae. aegypti* (Focks *et al.* 1993; Focks & Barrera 2006; Morin *et al.* 2015) | **B** | *T_min_* = 16.5 (14.0 – 18.3)  *T_max_* = 39.3 (37.7 – 40.0)  *q* = 3.03·10^-4^ (2.35 – 3.78·10^-4^)  optimum = 33.6°C |
| *bc* | Vector competence (transmission probability) |  |  | Did not use priors because they were asymmetric (B) while data were symmetric (Q) |
| *lf* | Adult lifespan (days) | *Ae. aegypti* (Beserra *et al.* 2009; Yang *et al.* 2009) | **Q** | *T_min_* = 6.4 (1.5 – 9.9)  *T_max_* = 39.3 (36.6 – 43.4)  *q* = 0.109 (0.0631 – 0.164)  optimum = 23.0°C |
| *PDR* | Parasite development rate (day)^-1^ | Dengue in *Ae. aegypti* (Davis 1932; McLean *et al.* 1974; Watts *et al.* 1987; Focks *et al.* 1993; Focks & Barrera 2006; Carrington *et al.* 2013; Tjaden *et al.* 2013); dengue in *Ae. Albopictus* (Xiao *et al.* 2014) | **B** | *T_min_* = 14.4 (4.1 – 19.5)  *T_max_* = 41.9 (38.1 – 44.9)  *q* = 1.05·10^-4^ (0.532 – 1.71·10^-4^)  optimum = 35.0°C |
| *EFD* | Fecundity (eggs per female per day) | *Ae. aegypti* (Beserra *et al.* 2009; Yang *et al.* 2009); *Ae. albopictus* (Calado & Navarro-Silva 2002); *Ae. krombeini* (Joshi 1996) | **Q** | *T_min_* = 13.4 (2.9 – 19.3)  *T_max_* = 33.9 (31.9 – 36.7)  *q* = 9.47·10^-3^ (3.47 – 19.0·10^-3^)  optimum = 28.8°C |
| *pRH* | Raft viability (probability of raft hatching) |  |  | No data for priors |
| *nLR* | Within-raft egg survival (number of larvae per raft) |  |  | No data for priors |
| *pLA* | Larval-to-adult survival (probability) | *Ae. aegypti* (Rueda *et al.* 1990; Tun-Lin *et al.* 2000; Kamimura *et al.* 2002; Westbrook *et al.* 2010; Eisen *et al.* 2014); *Ae. Albopictus* (Teng & Apperson 2000; Wiwatanaratanabutr & Kittayapong 2006; Delatte *et al.* 2009; Westbrook *et al.* 2010; Muturi *et al.* 2011); *Ae. triseriatus* (Jalil 1972) | **Q** | *T_min_* = 12.5 (11.2 – 13.6)  *T_max_* = 38.2 (37.3 – 39.2)  *q* = 5.31 (4.41 – 6.24)  optimum = 25.4 °C |
| *MDR* | Mosquito development rate (day)^-1^ | *Ae. aegypti* (Rueda *et al.* 1990; Tun-Lin *et al.* 2000; Kamimura *et al.* 2002); *Ae. albopictus* (Yee unpublished data) (Alto & Juliano 2001; Briegel & Timmermann 2001; Calado & Navarro-Silva 2002; Wiwatanaratanabutr & Kittayapong 2006; Delatte *et al.* 2009; Westbrook *et al.* 2010; Muturi *et al.* 2011; Ezeakacha 2015); *Ae. triseriatus* (Jalil 1972) | **B** | *T_min_* = 9.6 (5.3 – 12.7)  *T_max_* = 38.9 (37.7 – 41.4)  *q* = 7.06·10^-5^ (4.98 – 8.99·10^-5^)  optimum = 32.2°C |

**Figure 2-source data 2: Trait thermal response functions and data sources used to parameterize priors for data-informed trait thermal responses.** ‘Par.’ = model parameter. Fits were made with uniform priors. Asymmetrical responses fit with Brière function (**B**): B(*T*) = *qT*(*T* – *T_min_*)(*T_max_* – *T*)^1/2^; symmetrical responses fit with quadratic function (**Q**): Q(*T*) = -*q*(*T* – *T_min_*)(*T* – *T_max_*). Function coefficients (and 95% credible intervals) fit via Bayesian inference.
