# Supplementary material for: Temperature explains broad patterns of Ross River virus transmission
Source: eLife. 2018 Aug 28;7:e37762. doi: 10.7554/eLife.37762 (PMC6112853; doi:10.7554/eLife.37762)
Supplement: Figure 2—figure supplement 2—source data 1. — ‘Par.’=model parameter. Results are given for fits from data-informed priors. Asymmetrical responses fit with Brière function (B): B(T)= qT(T – Tmin)(Tmax – T)1/2; symmetrical responses fit with quadratic function (Q): Q(T) = -q(T – Tmin)(T – Tmax). Function coefficients (and 95% credible intervals) fit via Bayesian inference. This table also includes source data for Figure 2—figure supplement 3 and Figure 2—figure supplement 4. [file elife-37762-fig2-figsupp2-data1.docx]

| **Par.** | **Definition** | **Species (Sources)** | **Fit** | **Function Coefficients (95% CIs) & Optimal Temperature** |
| --- | --- | --- | --- | --- |
| *bc* | Vector competence (transmission probability) | MVEV in *Cx. Annulirostris* (Kay *et al.* 1989) | **NA** | Did not fit because no temperature signal |
| *PDR* | Parasite development rate (day)^-1^ | MVEV in *Cx. Annulirostris* (Kay *et al.* 1989) | **B** | *T_min_* = 12.8 (6.6 – 19.0)  *T_max_* = 41.9 (38.6 – 45.0)  *q* = 1.04·10^-4^ (0.641–1.55·10^-4^)  optimum = 34.8°C |
| *pLA* | Larval-to-adult survival (probability) | *Ae. camptorhynchus* (Barton & Aberton 2005) | **Q** | *T_min_* = 5.5 (1.9 – 10.1)  *T_max_* = 38.6 (35.7 – 42.3)  *q* = 3.02·10^-3^ (2.00 – 4.83·10^-3^)  optimum = 22.2°C |
| *pLA* | Larval-to-adult survival (probability) | *Ae. notoscriptus* (Williams & Rau 2011) | **Q** | *T_min_* = 9.1 (7.1 – 11.0)  *T_max_* = 36.2 (34.7 – 37.9)  *q* = 5.72·10^-3^ (4.37 – 7.32·10^-3^)  optimum = 22.8°C |
| *MDR* | Mosquito development rate (day)^-1^ | *Ae. camptorhynchus* (Barton & Aberton 2005) | **B** | *T_min_* = 9.5 (1.8 – 19.1)  *T_max_* = 38.8 (37.2 – 40.3)  *q* = 4.57·10^-5^ (2.50 – 8.29·10^-5^)  optimum = 32.2°C |
| *MDR* | Mosquito development rate (day)^-1^ | *Ae. notoscriptus* (Williams & Rau 2011) | **B** | *T_min_* = 9.6 (6.4 – 12.9)  *T_max_* = 38.7 (37.1 – 40.0)  *q* = 6.86·10^-5^ (5.43 – 8.56·10^-5^)  optimum = 32.0°C |

**Figure 2-figure supplement 2-source data 1: Trait thermal response functions and data sources for Murray Valley Encephalitis virus and additional vector species (*Ae. notoscriptus* and *Ae. camptorhynchus*).** ‘Par.’ = model parameter. Results are given for fits from data-informed priors. Asymmetrical responses fit with Brière function (**B**): B(*T*) = *qT*(*T* – *T_min_*)(*T_max_* – *T*)^1/2^; symmetrical responses fit with quadratic function (**Q**): Q(*T*) = -*q*(*T* – *T_min_*)(*T* – *T_max_*). Function coefficients (and 95% credible intervals) fit via Bayesian inference. This table also includes source data for Figure 2-figure supplement 3 and Figure 2-figure supplement 4.
